# Characterization and genome analysis of novel *Klebsiella pneumoniae* phage vbKpUKJ_2 isolated from hospital sewage water

**DOI:** 10.1186/s12866-025-03813-y

**Published:** 2025-02-26

**Authors:** Kamran Ahmad Mirza, Tinatini Tchatchiashvili, Mike Marquet, Sandor Nietzsche, Mathias W. Pletz, Oliwia Makarewicz

**Affiliations:** 1 Institute of Infectious Diseases and Infection Control, Jena University Hospital, Friedrich-Schiller-University Jena, Am Klinikum 1, 07747 Jena, Germany; 2 Center for Electron Microscopy, Jena University Hospital, Friedrich-Schiller-University Jena, 07743 Jena, Germany; 3 Leibniz Center for Photonics in Infection Research, 07747 Jena, Germany

**Keywords:** Bacteriophage therapy, *Klebsiella pneumoniae*, Multidrug-resistant bacteria, Phage characterization, Genome analysis

## Abstract

**Introduction:**

Multidrug-resistant (MDR) *Klebsiella pneumoniae* is a critical healthcare challenge due to its extensive resistance to antibiotics and role in causing severe infections. Bacteriophages offer a promising alternative for targeting MDR pathogens. This study characterizes a novel phage, vbKpUKJ_2, isolated from hospital sewage water, against clinical *K. pneumoniae* isolates.

**Methods:**

Phage vbKpUKJ_2 was isolated and purified using the double agar overlay method. Host range and sensitivity were tested against 40 clinical *K. pneumoniae* isolates using growth inhibition assays. Morphological characterization was performed using transmission electron microscopy (TEM). Genomic analysis was conducted to evaluate the absence of antibiotic resistance genes and determine phylogenetic relationships. Stability assays assessed the phage’s thermal and pH tolerance.

**Results:**

Phage vbKpUKJ_2 demonstrated broad range activity against clinical *K. pneumoniae* isolates. TEM revealed it belongs to the Drexlerviridae family. Genomic analysis confirmed the absence of antibiotic resistance genes and identified conserved functional regions shared with related phages. vbKpUKJ_2 exhibited broad pH stability (pH 4–10) and thermal stability between 30 °C and 60 °C. The one-step growth curve indicated rapid lytic activity, with a burst size of 323 phage particles per cell.

**Conclusion:**

vbKpUKJ_2 shows promising therapeutic potential against MDR *K. pneumoniae*. Its stability, absence of resistance genes, and rapid lytic cycle highlight its suitability for inclusion in phage therapy protocols, particularly as part of combination therapies targeting MDR infections.

**Supplementary Information:**

The online version contains supplementary material available at 10.1186/s12866-025-03813-y.

## Key summary points

• A novel bacteriophage, vbKpUKJ_2, was isolated from hospital sewage and characterized for its therapeutic potential against multidrug-resistant (MDR) *Klebsiella pneumoniae* isolates.
• vbKpUKJ_2 demonstrated activity against 43.5% of tested clinical *K. pneumoniae* isolates, with no latent phase and rapid lytic activity, achieving a burst size of 323 phage particles per cell.
• Genomic analysis confirmed the absence of antibiotic resistance genes, ensuring safety for therapeutic use, and placed vbKpUKJ_2 in the Drexlerviridae family, closely related to other lytic phages.
• Stability assays showed that vbKpUKJ_2 remains active across a broad pH range (4– 10) and temperatures of 30–60 °C, supporting its robustness for diverse applications.
• These findings highlight vbKpUKJ_2 as a promising candidate for phage therapy, particularly in combination treatments targeting MDR *K. pneumoniae* infections.

**Supplementary Information:**

The online version contains supplementary material available at 10.1186/s12866-025-03813-y.

## Introduction

*Klebsiella pneumoniae* is an opportunistic pathogen, causing infections such as pneumonia, urinary tract and blood stream infections. Particularly in hospitalized and immunocompromised patients these infections can progress to sepsis and septic shock [1]. It poses a considerable challenge in healthcare settings, where its ability to cause hospital-acquired infections such as ventilator-associated pneumonia in ICU-patients is of great concern [2]. The gastrointestinal tract serves as a primary reservoir for *K. pneumoniae* colonization, including carbapenem-resistant strains [3]. Colonization with carbapenem-resistant *K. pneumoniae* significantly increases the risk of systemic infections and associated mortality, particularly in high-risk patient populations, such as those in critical care, post-surgical patients, or individuals with immunosuppression [4]. Evidence suggests that the intestinal burden of carbapenem-producing *K. pneumoniae* is a critical factor for the subsequent risk of infection in hospitalized patients [5]. According to 2017 and 2024 report of World Health Organization (WHO), *K. pneumoniae* has been recognized as a critical pathogen due to its high prevalence of resistance to commonly used third-generation cephalosporins and last-resort carbapenem antibiotics [6, 7]. *K. pneumoniae* strains have become highly prevalent in both, community-acquired and nosocomial infections, contributing to the global rise in multidrug-resistant (MDR) pathogens [8], highlighting a critical global demand for alternative therapeutic strategies [9].

Bacteriophages (phages) are viruses that infect bacteria and offer a promising alternative to traditional antibiotics. Phages exhibit an extremely high specificity, often down to the strain level, without disrupting the host’s essential microbiota [10]. The selective activity of phages makes them ideal candidates for combating MDR bacteria while reducing the risk of antibiotic-induced dysbiosis and secondary infections. In our recent studies, phage treatment was successfully used in a *Galleria mellonella* gut colonization model with *K. pneumoniae* and significantly reduced bacterial loads, highlighting their potential as a viable treatment option [11].

In this study, we isolate and characterize a novel *K. pneumoniae* phage, vbKpUKJ_2, isolated from sewage water at Jena University Hospital, Germany. The phage was extensively characterized through phenotypic, genomic analysis and host range determination to assess its efficacy against 40 clinical *K. pneumoniae* isolates in vitro.

## Materials and methods

### Phage isolation, purification, and storage

Phage vbKpUKJ_2 was isolated from sewage water of the Jena University Hospital using the double agar overlay technique. The sewage sample was centrifuged at 10,000 rcf for 20 min, and the supernatant was collected and sterile-filtered using a 0.2 μm filter. The overnight culture of *K. pneumoniae* isolate Kp419614 was grown in Luria-Bertani (LB) broth (Oxoid Deutschland GmbH, Wiesel, Germany) at 37 °C with shaking at 150 rpm. The filtered supernatant was then incubated with an overnight culture with 10^8^ CFU/mL of the *K. pneumoniae* isolate Kp419614 in a shaking incubator at 120 rpm at 37 °C. After overnight incubation, the culture was centrifuged at 10,000 rcf for 20 min to pellet bacterial debris. The supernatant was filtered through a sterile 0.2 μm membrane filter to ensure sterility. Serial dilutions of the filtered supernatant were prepared, and were used for the double-layer agar assay to detect and quantify phage. Luria-Bertani (LB) agar (Oxoid Deutschland GmbH, Wiesel, Germany) plates were freshly prepared and used as the base agar, with a freshly prepared 0.5% LB agar top layer for the double-layer agar assay [12]. Plates were incubated overnight and plaques were examined visually, and individual plaques were selected based on size and morphology for further characterization.

To ensure the purity of the phage, selected plaques were picked using 1 mL pipette tips and subjected to three rounds of proliferation using the double agar overlay method. Following the purification process, the phage stock was titered using a serial dilution to reach a concentration of at least 10^10^ plaque-forming units per milliliter (PFU/mL). Once the desired titer was achieved, the purified phage was cryopreserved in cryotubes containing 10% glycine. The cryotubes were labeled with the phage titer and stored at -180 °C for long-term preservation.

### Scanning of host range

To determine the host spectrum of the isolated phage, growth curve experiments were conducted to assess phage infection against 40 clinical isolates of *K. pneumoniae* with different antimicrobial resistance phenotypes, which were previously collected in studies approved by the ethical committee of Jena University Hospital (approval numbers 3852/07–13 and 3694-02/13) (Supplementary Data, Table S1). Growth curve experiments were conducted with a multiplicity of infection (MOI) of 1, corresponding to an initial concentration of 10^8^ PFU/mL for the phage and 10^8^ CFU/mL for the bacterial cells. Growth was continuously monitored over a 10 h by measuring optical density (OD) at 600 nm at 37 °C and shaking using an Infinite 200 PRO microplate reader (Tecan Group AG, Männedorf, Switzerland) and experiment was conducted with three technical and biological replicates (*n* = 3). Bacterial isolates were categorized based on growth inhibition as “susceptible” if inhibition persisted for 5 h or more, “intermediate susceptible” if inhibition lasted for less than 5 h, and “resistant” if no inhibition was observed compared to the control over the 10 h period.

### Transmission electron microscopy (TEM) for morphological characterization

The morphological characteristics of phage vbKpUKJ_2 were analyzed using negative staining technique utilizing a transmission electron microscope (TEM) model EM 900 (Zeiss, Oberkochen, Germany) at an accelerating voltage of 80 kV and a magnification of 250,000x. Therefore, a droplet of purified phage solution with a concentration of at least 10^9^ PFU/mL was placed on a TEM grid (type R2/1 + 2nm_C_Cu400, Quantifoil, Jena, Germany) for 10 min to allow adsorption of phage particles onto the grid. After removing the phage droplet the grid was placed on a staining droplet of 2% (w/v) uranyl acetate for 1 min. Finally, the grid was washed twice with purified water, air-dried, and studied by TEM. For image recording, a 2 K slow scan CCD camera (TRS, Moorenweis, Germany) was used.

### Determination of optimal multiplicity of infection

The MOI, defined as the ratio of phage particles to host bacterial cells at the initiation of infection, was established by preparing inoculations across a range of MOIs in sterile LB broth. Phage solutions and bacterial cultures were prepared separately, with 100 µL of each, a phage suspension (concentrations of 10^8^, 10^7^, 10^6^, 10^5^, 10^4^, 10^3^ PFU/mL) and a bacterial suspension at 10^8^ CFU/mL added to 800 µL of sterile LB broth. This setup yielded MOIs of 1, 0.1, 0.01, 0.001, 0.0001, and 0.00001, respectively. The mixtures were incubated at 37 °C with shaking at 150 rpm for 5 hours to facilitate phage-host interaction and infection. Post-incubation, the samples were centrifuged at 10,000 rcf for 10 min to pellet bacterial debris. The supernatant was then carefully collected and passed through a 0.2 μm sterile filter to eliminate residual bacterial cells, ensuring sterility in the phage-containing supernatant. Phage titers in the filtered supernatant were determined using the standard double agar overlay assay, with each experiment conducted with three technical and biological replicates (*n* = 3). The MOI yielding the highest phage titer was designated as the optimal condition for phage propagation.

### One-step growth curve analysis

The host bacterium, Kp419614, was cultured in LB broth until it reached the logarithmic growth phase. Subsequently, the phage was added to the bacterial culture at a MOI of 0.01 (10^8^ CFU/mL bacteria and 10^6^ PFU/mL phage), and the mixture was incubated at 37 °C for 10 min to allow adequate time for phage adsorption onto the host cells. After the adsorption period, the culture was centrifuged at 10,000 rcf for 10 min to pellet the bacterial cells with adsorbed phages [13]. The supernatant was carefully discarded, and the bacterial pellet was resuspended in fresh LB broth to continue the experiment. Samples (100 µL) were taken at defined intervals of 0, 10, 20, 30, 40, 60, 90, 120, and 150 min post-resuspension. Each sample was subjected to 10-fold serial dilution in 1 x PBS, and the phage titer was determined using the double agar overlay method. The number of PFU/mL was recorded at each time point and experiment was conducted with three technical and biological replicates (*n* = 3).

### Thermal stability and pH tolerance of phages

The thermal stability of the phages was assessed by incubating 10^6^ PFU/mL phage samples in 1 x PBS buffer at 30, 40, 50, 60, 70, and 80 °C for 60 min. After incubation, the phage titer was determined using the double-layer agar method. The experiment was conducted with three technical and biological replicates (*n* = 3) for each temperature. To evaluate the tolerance of 10^6^ PFU/mL phages to different pH levels, 1 x PBS with varying pH values of 2, 4, 6, 8, 10, and 12 were prepared. The buffer solutions were sterilized by autoclaving at 121 °C for 20 min and then cooled to room temperature. A 100 µL aliquot of the phage solution was mixed with 900 µL of each pH buffer and incubated at room temperature for 1 h. Phage titers in each pH treatment were determined using the double agar overlay method. The experiment was conducted with three technical and biological replicates (*n* = 3).

### Library preparation

Phage capsid was lysed with proteinase K and phage DNA isolation was performed using the ZymoBIOMICS DNA Miniprep Kit (Zymo Research Europe GmbH, Freiburg, Germany) according to the protocol, specifically optimized for long-read sequencing. Quantification of the extracted DNA was conducted using the dsDNA HS assay on a Qubit fluorometer (Invitrogen, USA) to ensure accurate DNA concentration for library preparation. DNA samples were then size selected by cleaning up with 0.45x volume of Ampure XP buffer (Beckman Coulter, Brea, CA, USA), which helped in removing short fragments, followed by elution in 50 µL EB buffer (Qiagen, Hilden, Germany). The library for sequencing was prepared using 1 µg of DNA and the SQK-LSK114 kit (Oxford Nanopore Technologies, Oxford, UK), following the manufacturer’s protocol to ensure optimal library quality and yield.

### Nanopore sequencing and basecalling

Sequencing was conducted using the Oxford Nanopore Technology (ONT) platform to generate long-read data. Basecalling of raw reads was performed with the Dorado software (v7.3.11) (Oxford Nanopore Technologies), using the high super accuracy basecalling model to ensure maximal read accuracy. Post-basecalling, reads were assembled and polished through a pipeline involving Flye (v2.9.3) [14] for assembly, followed by polishing steps using Medaka (v1.11.3) and Racon (v1.4.20) to improve the accuracy and quality of the final consensus sequence. These steps ensured high-quality assemblies for downstream analyses.

### Genome sequencing and bioinformatics analysis

Open reading frames (ORFs) and the complete phage genome were predicted using PHASTEST (http://www.phastest.ca) [15] and What the Phage [16]. Antimicrobial resistance genes were identified using ARMFinder tool [17]. Further, the genome annotation of vbKpUKJ_2 was refined using BLAST.

The whole genome of phage vbKpUKJ_2 was analyzed using the Mauve genome alignment tool [18]. A phylogenetic tree was constructed with MEGA11 [19] to assess evolutionary relationships. Additionally, a proteome tree based on the whole genome sequence was generated using VipTree (https://www.genome.jp/viptree) [20].

### Statistical and analytical tool

Microsoft Excel (Microsoft Corporation, Redmond, USA) and GraphPad Prism 10 (GraphPad Software Inc., San Diego, USA) were employed for data calculations, statistical analyses, and visualization. All experiments were conducted in biological triplicates, with results expressed as the mean ± standard deviation. For rate analysis, the first derivative was calculated based on discrete time points. Assuming x and y represent data arrays and x′ and y′ the derivative results, with t denoting time points:

$$\:\text{x}{\prime\:}\left[\text{t}\right]=\left(\text{x}\right[\text{t}+1]+\text{x}[\text{t}\left]\right)\:/\:2\:$$

$$\:\text{y}{\prime\:}\:\text{a}\text{t}\:\text{x}{\prime\:}\left[\text{t}\right]=\left(\text{y}\right[\text{t}+1]-\text{y}[\text{t}\left]\right)\:/\:\left(\text{x}\right[\text{t}+1]-\text{x}[\text{t}\left]\right).$$

## Results

### Morphological characteristics and host range

**Fig. 1:**
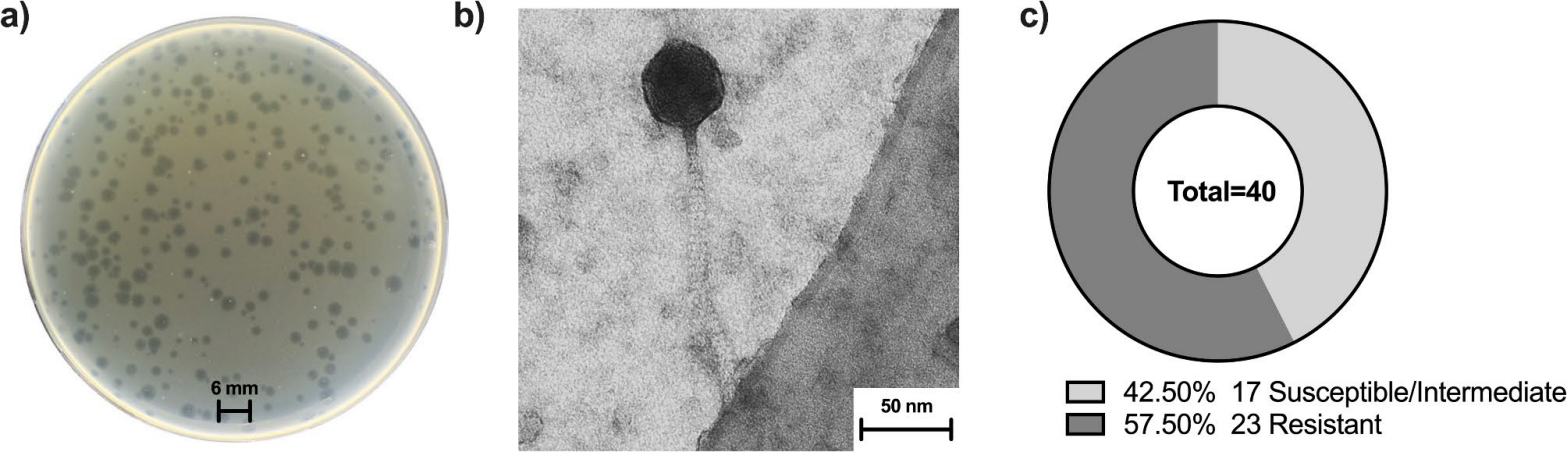
(**a**) Plaques formed by phage vbKpUKJ_2 on *K. pneumoniae* Kp419614 host using the double agar overlay method. The plaques are clear with an average diameter of approximately 3 mm, indicating a lytic phage. Scale bar: 3 mm. (**b**) Transmission electron microscopy (TEM) image of phage vbKpUKJ_2. The phage was characterized by an icosahedral head (~ 50 nm diameter) and a long, flexible, non-contractile tail (~ 200 nm). Scale bar: 50 nm. (**c**) Host range analysis of phage vbKpUKJ_2 against 40 *K. pneumoniae* clinical isolates. The pie chart represents the proportion of isolates classified as susceptible/intermediate (43%) and resistant (57%) to phage infection. This distribution indicates selective efficacy of vbKpUKJ_2 against certain *K. pneumoniae* strains

Phage vbKpUKJ_2 was isolated from hospital sewage water and formed clear, circular plaques on the double agar overlay assay (Fig. 1a), with an average diameter of approximately 3 mm. The sharp, clear plaques suggest that vbKpUKJ_2 is a lytic phage, efficiently infecting and lysing its ESBL producing *K. pneumoniae* host, Kp419614. Transmission electron microscopy (TEM) imaging (Fig. 1b) revealed that phage vbKpUKJ_2 characterized by an icosahedral head measuring approximately 50 nm in diameter and a long, flexible, non-contractile tail measuring about 200 nm. The repeated isolation of uniform plaques with similar morphology and size further supports the purity and stability of the phage preparation.

Phage vbKpUKJ_2 was tested against 40 clinical isolates of *K. pneumoniae* (Fig. 1c and Supplementary data, Table S1). The results revealed varying levels of susceptibility, with isolates classified as susceptible, intermediate susceptible, or resistant to phage infection. Out of the 40 tested isolates, 11 were susceptible to phage vbKpUKJ_2. Six isolates showed intermediate susceptibility, with partial lysis observed (Supplementary data, Table S1). In contrast, 23 isolates were resistant to vbKpUKJ_2, showing no signs of lysis (Supplementary data, Table S1).

### Optimal MOI, one-step growth, thermal and pH stability

**Fig. 2:**
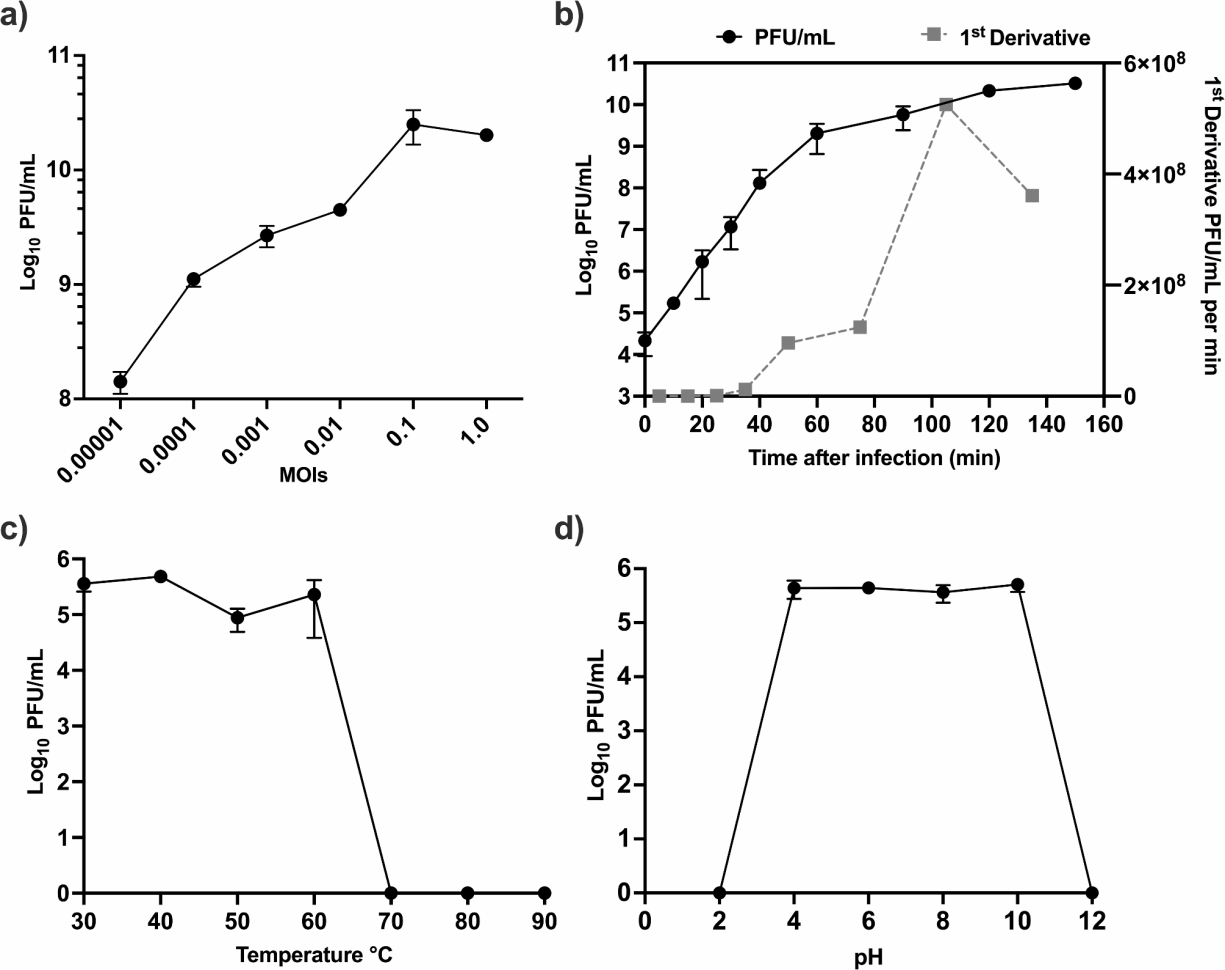
(**a**) Phage vbKpUKJ_2 proliferation at varying MOIs. At an MOI of 1 (10⁸ PFU/mL phage, 10⁸ CFU/mL host), the phage reached 2.02 × 10¹⁰ PFU/mL, indicating efficient infection. An MOI of 0.1 yielded 2.50 × 10¹⁰ PFU/mL, suggesting effective amplification with abundant hosts. Lower MOIs (0.0001, 0.00001) produced reduced yields (1.12 × 10⁹, 1.42 × 10⁸ PFU/mL), showing limited final output due to lower initial phage concentrations. (**b**) One-step growth curve of phage vbKpUKJ_2 infecting *K. pneumoniae* strain Kp419614, showing immediate titer increase post-infection with peak production at 105 min and a burst size of 323 phage particles per cell. The experiment was repeated thrice (*n* = 3). The data points represent mean and the error bar represent the standard deviation. Temperature (**c**) and pH (**d**) stability of phage vbKpUKJ_2. The phage remained active between 30 °C and 60 °C, with minimal loss in titer, but showed significant reduction in titer and almost complete inactivation at 70 °C. The pH stability test revealed that vbKpUKJ_2 was highly stable across a broad pH range of 4 to 10, with a noticeable decline in activity at pH values below 4 and above 10. The experiment was repeated thrice (*n* = 3). The data points represent mean and the error bar represent the standard deviation

The proliferation of phage vbKpUKJ_2 was evaluated at different MOIs to determine the impact of varying initial phage concentrations on the final phage yield (Fig. 2a). At an MOI of 1, where the initial concentrations of both the phage and host were 10^8^ PFU/mL and 10^8^ CFU/mL respectively, the phage vbKpUKJ_2 achieved a high final concentration of 2.02 × 10^10^ PFU/mL. This result suggests a highly efficient infection process when the phage and host are present in equal concentrations, leading to robust phage proliferation. At an MOI of 0.1, with an initial phage concentration of 10^7^ PFU/mL, the phage yielded an even higher final concentration of 2.50 × 10^10^ PFU/mL. This indicates that a lower phage-to-host ratio can still result in efficient phage amplification, likely due to the abundant availability of susceptible host cells. Contrary, at the lowest MOIs tested, 0.0001 and 0.00001, the final phage concentrations were 1.12 × 10^9^ PFU/mL and 1.42 × 10^8^ PFU/mL, respectively. While phage replication still occurred, the lower MOIs resulted in a reduced final phage output, indicating that insufficient initial phage concentration limits overall proliferation.

The one-step growth curve analysis of phage vbKpUKJ_2 demonstrated rapid replication kinetics, with no observable latent period. The phage titer increased almost immediately following infection, suggesting efficient adsorption and swift lysis of the Kp419614 host cells (Fig. 2b). The burst size of phage vbKpUKJ_2 was determined using two approaches. Based on the plateau method, the burst size was calculated as 323 ± 88 phage particles per cell. Alternatively, using the first derivative method, it was calculated as 5 phage particles per cell. Analysis of the first derivative of the growth curve revealed peak phage production occurring at 105 min post-infection. The plateau phase indicated that the maximal phage concentration had been reached, marking the completion of the lytic cycle for infected cells. The infection parameters included an incorporation rate of 8.50 × 10^− 3^ phages/PFU, alongside an infected bacteria count of 8.50 × 10^− 6^ bacteria/CFU. These metrics further underscore vbKpUKJ_2’s efficacy in initiating a rapid lytic cycle, which, when combined with the absence of a latent period, highlights its potential for swift and effective bacterial clearance.

The stability of phage vbKpUKJ_2 was assessed across a range of temperatures (Fig. 2c). The phage remained active between 30 °C and 60 °C, with minimal loss in titer. At 70 °C and above, there was a significant reduction in phage titer, with almost complete inactivation. This suggests that vbKpUKJ_2 is stable at wide range of temperatures but loses viability at higher temperatures such as 70 °C. The pH stability test demonstrated that phage vbKpUKJ_2 was highly stable across a broad pH range (Fig. 2d). The phage remained fully active between pH 4 and pH 10. At more extreme pH values, such as below pH 4 and above pH 10, a significant reduction in phage activity was noted.

### Genome features and annotation

Phage vbKpUKJ_2 genome was analyzed for the presence of antibiotic resistance genes, and no resistance genes were detected (Supplementary data, Table S2). This is a critical feature for its application in phage therapy, as the absence of resistance genes reduces the risk of horizontal gene transfer, ensuring that the phage does not contribute to the spread of antibiotic resistance. The negative results for various antibiotics, including amikacin, β-lactams, aminoglycosides, and many others are mentioned.

**Fig. 3:**
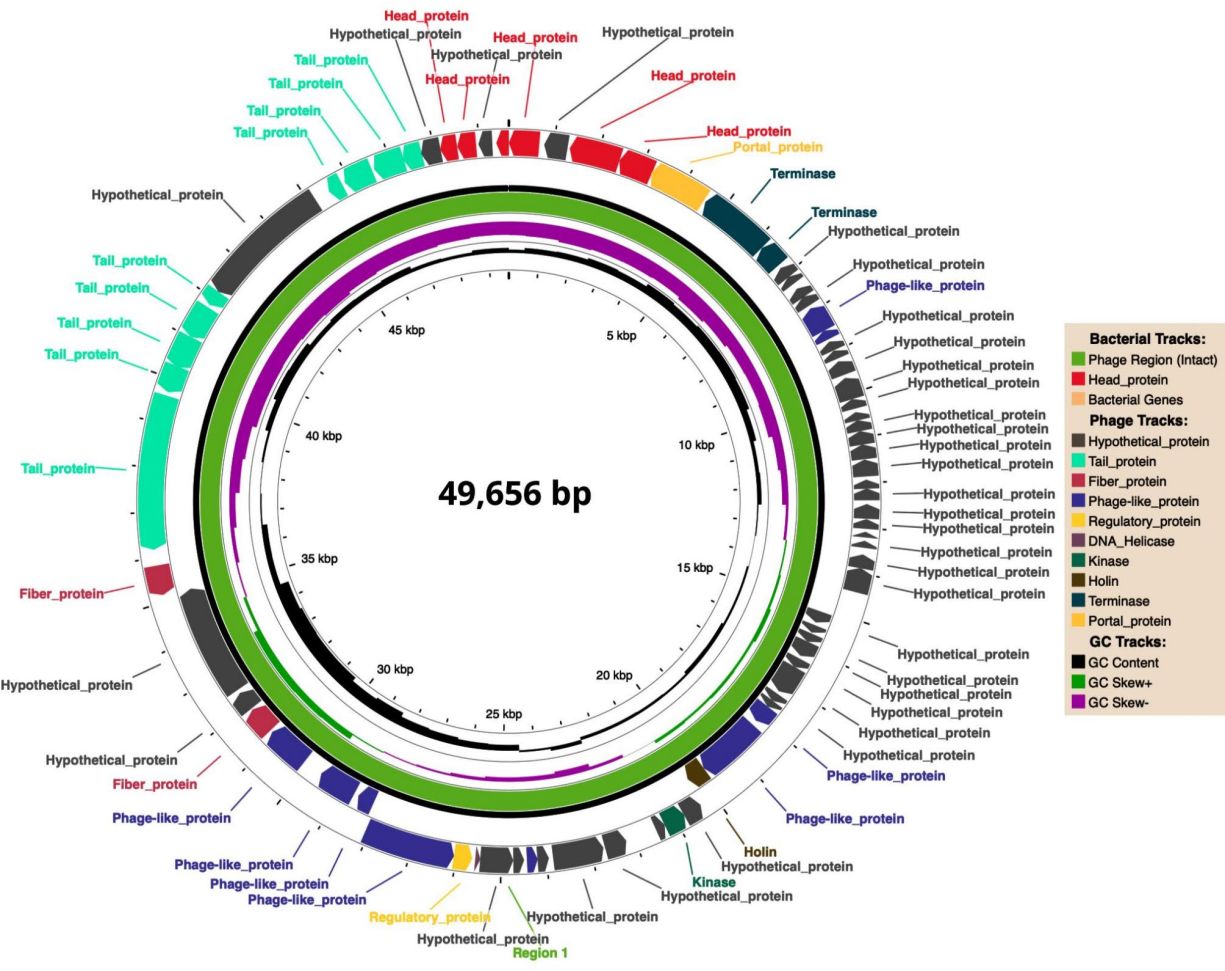
Circular genome map of phage vbKpUKJ_2, showing a genome size of 49,656 base pairs. The outermost track represents the functional annotation of the phage genome, including structural proteins such as tail proteins (green), fiber proteins (light red), and head proteins (strong red). Hypothetical proteins are indicated in gray, while regulatory and phage-like proteins are marked in yellow and blue, respectively. The inner tracks display the GC content and GC skew, providing insights into the genomic structure. Key proteins such as terminase, DNA helicase, and kinases are highlighted, emphasizing the phage’s structural and functional complexity. Genome annotation and visualization were performed using PHASTEST [15]. No bacterial genes were identified in the genome annotation. The slight orange legend indicating bacterial genes is by default in the software’s output and cannot be changed

The whole genome of phage vbKpUKJ_2 was sequenced and analyzed. The genome consists of several open reading frames (ORFs) encoding hypothetical proteins, transcriptional regulators, structural proteins, and lysis-associated proteins (Fig. 3). Notably, key structural proteins such as the tail fiber, major head, and terminase proteins were identified. A detailed list of predicted ORFs and their functions is presented in the attached file (vbKpUKJ_2_genes_prediction). Additionally, lysis-related proteins such as lysozymes and holins were identified, emphasizing the phage’s lytic capacity. However, no prediction for endolysin was observed.

**Fig. 4:**
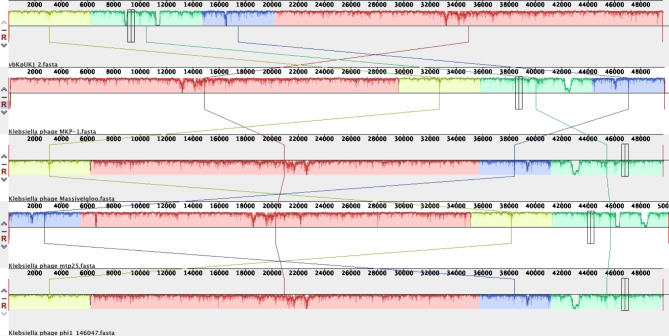
Mauve alignment of *Klebsiella* phage vbKpUKJ_2 with closely related phages (*Klebsiella* phages MKP-1, Massivegloo, mtp25, and phi1_146047). Colored blocks (Locally Collinear Blocks) represent conserved regions, with blocks below the axis indicating inversions. Structural rearrangements, including inversions and translocations, are observed in mid-genome regions, while terminal regions show higher conservation with minor variations

A comparative genomic analysis of phage vbKpUKJ_2 against closely related *Klebsiella* phages MKP-1, Massivegloo, mtp25, and phi1_146047 (Fig. 4) was performed using Mauve alignment. This analysis revealed extensive conservation in genomic regions, particularly in the central and terminal portions of the genomes, suggesting that these areas are likely critical for core phage functions such as replication and packaging. However, significant variability was observed in the early genomic regions of vbKpUKJ_2, with noticeable gaps in alignment, possibly indicating unique sequences or structural variations specific to vbKpUKJ_2. Furthermore, multiple structural rearrangements, including inversions and translocations, were evident, especially in the mid-genome and terminal regions. These structural differences highlight evolutionary divergence between vbKpUKJ_2 and its closely related phages, suggesting that vbKpUKJ_2 may have undergone distinct evolutionary pressures leading to these genomic alterations.

### Genome comparison and phylogenetic tree

**Fig. 5:**
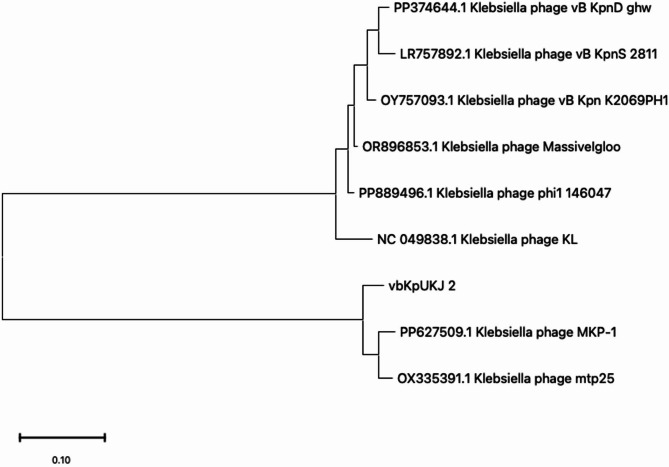
Maximum likelihood phylogenetic tree based on the complete genomes of *Klebsiella* bacteriophages, including vbKpUKJ_2. The tree demonstrates the evolutionary relationships between vbKpUKJ_2 and closely related phages, with vbKpUKJ_2 clustering closely with *Klebsiella* phages MKP-1 and mtp25, indicating a recent common ancestor. More distantly related phages, such as phi1_146047 and Massivegloo, form separate clusters with longer branch lengths, suggesting greater evolutionary divergence. The scale bar represents the number of substitutions per site, providing an indication of evolutionary distance

A maximum likelihood phylogenetic tree was constructed to assess the evolutionary relationships between *Klebsiella* phage vbKpUKJ_2 and its closest relatives identified via BLAST. The analysis revealed that vbKpUKJ_2 is closely related to *Klebsiella* phages MKP-1 and mtp25, forming a distinct clade with relatively short branch lengths, suggesting a recent common ancestor (Fig. 5). In contrast, more distantly related phages, such as phi1_146047 and Massivegloo, formed separate clusters with longer branch lengths, indicating greater evolutionary divergence. The phylogenetic clustering aligns with the genomic similarities observed in the Mauve alignment and highlights the genomic diversity among *Klebsiella* phages. These results suggest that vbKpUKJ_2 and its closely related phages share significant genomic features.

**Fig. 6:**
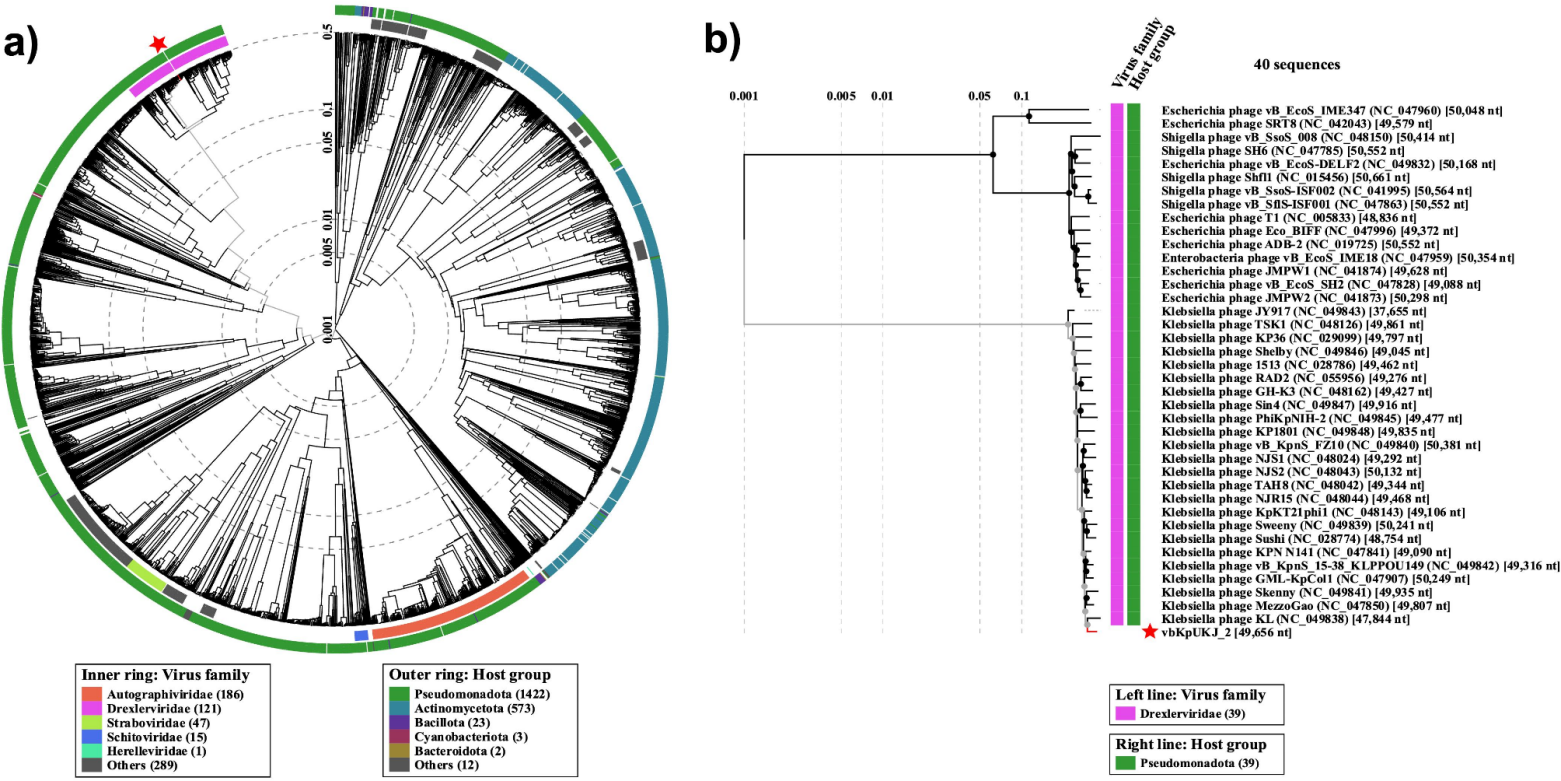
(**a**) Circular phylogenetic tree of bacteriophages based on whole proteome similarity. The *Klebsiella* phage vbKpUKJ_2, marked by the red star, is positioned among a diverse set of bacteriophages. Color coding represents different viral families: purple for Drexlerviridae, orange for *Autographiviridae*, and blue for *Schitoviridae*. vbKpUKJ_2 clusters within the *Drexlerviridae* family, indicating its evolutionary proximity to other members of this family, while other families form distinct clusters, illustrating their evolutionary divergence. (**b**) The rectangular phylogenetic tree is a zoomed-in subset of the *Drexlerviridae* family from the circular tree, focusing on vbKpUKJ_2 (marked by the red star) and its closest relatives. The short branch lengths suggest close evolutionary relationships and recent common ancestry, further supporting its classification within *Drexlerviridae*

A detailed proteome phylogenetic analysis was conducted using VipTree to examine the evolutionary relationships of *Klebsiella* phage vbKpUKJ_2 and its related phages. The analysis, based on whole proteome similarity, placed vbKpUKJ_2 firmly within the *Drexlerviridae* family, as indicated by its location in the purple clade in the circular proteomic tree (Fig. 6a). The tree showcases the evolutionary distance between phage families, with vbKpUKJ_2 clustering closely with other *Drexlerviridae* phages. In contrast, phages from other families, such as *Autographiviridae* (represented in orange) and *Schitoviridae* (represented in blue), form distinct clusters in the circular tree, showing much greater evolutionary divergence from vbKpUKJ_2. This divergence is likely due to differences in host specificity, genomic architecture, or environmental adaptation.

The rectangular phylogenetic tree provides a more zoomed view of vbKpUKJ_2 and its close relatives within *Drexlerviridae* (Fig. 6b). The shorter branch lengths between vbKpUKJ_2 and other members of the family suggest a high degree of genomic and proteomic similarity, reinforcing the idea that these phages have evolved from a common ancestor relatively recently. This grouping within *Drexlerviridae* also points to shared structural or functional elements that are key to their evolutionary success and host specificity, particularly toward *Klebsiella* species.

## Discussion

Wastewater, particularly from hospitals, is a highly selective environment due to increased concentration of antibiotics. It serves as a reservoir for multidrug-resistant bacteria [21], especially *Enterobacteriaceae* such as *K. pneumoniae* that are often resistant to the commonly used antibiotics. Since phages are natural companions of bacteria, the likelihood increases to find phages targeting *K. pneumoniae* in wastewater [22].

Based on morphological, genomic, and phylogenetic analyses, we could assign the isolated phage vbKpUKJ_2 as a member of the *Drexlerviridae* family, which are known for their ability to infect Gram-negative bacteria such as *K. pneumoniae* [23]. *Drexlerviridae* family phage (e.g. T1 phage) are non-enveloped and possess a head-tail structure with a Sipho-type morphology, featuring an icosahedral head approximately 60 nm in diameter and a non-contractile, highly flexible tail approximately 150 nm long and 8 nm wide, ending in four short, kinked terminal fibers. The TEM-morphology of the phage vbKpUKJ_2 aligns well with the *Drexlerviridae* family. Genomic sequencing revealed conserved genomic elements shared with other *Klebsiella* phages, such as phi1_146047, mtp25, MKP1, and Massivegloo. Comparative genomic analysis using Mauve alignment highlighted these conserved regions, particularly in essential functional genes, while unique regions likely contribute to its host specificity. Phylogenetic analysis further supported vbKpUKJ_2 close evolutionary relationships with phages MKP-1 and mtp25, suggesting a shared evolutionary history. Additionally, proteome analysis comparing vbKpUKJ_2 protein profiles with other related phages revealed its classification within the *Drexlerviridae* family, demonstrating its evolutionary proximity to other clinically significant phages and providing a solid foundation for its potential therapeutic application. The genomic analysis of vbKpUKJ_2 aligns with findings from similar studies on lytic *K. pneumonia*e phages, such as vB_KpnM-VAC66 and vB_KpnM-VAC13, which also demonstrated the absence of antibiotic resistance genes and the presence of conserved structural proteins essential for lytic activity [24]. These conserved genomic features underscore the potential of vbKpUKJ_2 as a safe and effective candidate for phage therapy. In terms of infection mechanisms, phage structural proteins, such as tail fibers and capsid proteins, play a crucial role in host specificity and infection efficiency [25]. The presence of holin and lysozyme genes indicate a lytic lifecycle as these proteins facilitate bacterial cell wall degradation and host cell lysis during the phage infection process [26]. The presence of putative tail depolymerase, which is associated with enzymatic degradation of capsular polysaccharides and might be the reason of halo phenotype observed in plaque assays. However, further experimental validation is needed to confirm its depolymerase activity. The identification of similar structural elements in vbKpUKJ_2 suggests that its host recognition and lytic capabilities are consistent with other well-characterized therapeutic phages.

The stability of phage vbKpUKJ_2 across a broad range of temperatures (30–60 °C) and pH conditions (pH 4–10) indicates it has robust resistance to harsh conditions, a valuable feature for formulating phage preparations for oral, local, or even systemic application. The presence of genes encoding DNA helicase, DNA repair exonuclease, and DNA methyltransferase has likely contributed to maintaining genomic integrity, while the structural proteins such as the major capsid protein, portal protein and intrinsic physiochemical properties of capsid and tail structure are likely involved in maintaining capsid integrity under stress conditions. While the pH stability of vbKpUKJ_2 suggests potential for survival in environments like the intestines, direct passage through the highly acidic stomach (where pH can fall below 4) may require additional encapsulation techniques to ensure delivery to the gut. The physiochemical stability supports the phage’s potential for gut decolonization of *K. pneumoniae* in patients at high risk of infection, such as those preparing for invasive procedures. Additionally, vbKpUKJ_2 could serve in local therapeutic applications on infected tissues or as a prophylactic adjunct to standard treatments in healthcare environments to minimize pathogen persistence and spread without disrupting the host’s beneficial microbiota.

Phage vbKpUKJ_2 demonstrated lytic activity against 43.5% of the tested, non-clonally related, clinical *K. pneumoniae* isolates, suggesting it may be effective against a relevant proportion of *K. pneumoniae* strains, including some MDR variants. Notably, the phage exhibited no latent phase and showed rapid lysis, with a maximum lytic efficiency of 5.25 × 10^8^ PFU/mL per minute. This fast lytic activity confirms its virulence, further supporting its potential as a therapeutic candidate, although its efficacy may be limited by the strain-specific nature of the phages. However, we used randomly selected clinical *K. pneumoniae* isolates representing the prevalence in our hospital rather than selected serotypes, as serotyping is not part of our routine diagnostic pipeline. Therefore, it is possible that vbKpUKJ_2 may exhibit specificity for certain serotypes, a factor that should be explored in future studies to optimize its therapeutic potential. This study did not assess the frequency of phage-resistant mutant emergence, a critical factor for long-term efficacy, which warrants investigation in future studies. Personalized bacteriophage therapy has been shown to be highly effective when phages are tailored to specific bacterial strains [27]. Studies on clinical phage therapy outcomes highlight the importance of ensuring phages have activity against relevant clinical isolates, as well as the absence of resistance genes [27]. The broad host range and robust lytic activity of vbKpUKJ_2, coupled with its stability across diverse conditions, position it as a promising candidate for addressing multidrug-resistant *K. pneumoniae* infections.

Phage cocktails against *K. pneumoniae* tested in vitro often mix different phage families (such as *Podoviridae* (e.g., T7) and *Myoviridae* (e.g., T4)) [28] to broaden the spectrum of activity and avoid competition for receptors. Mixing phages with different and known receptor specificities reduces the likelihood of cross-resistance and enhances effectiveness against diverse strains, including MDR variants [29]. However, one of the limitations of the phage cocktail is the potential competition among phages for the same bacterial receptors, which could reduce the overall efficacy of the cocktail. Nonetheless, incorporating vbKpUKJ_2, a phage from the *Drexlerviridae* family, into such a cocktail could complement phages from other families, improving therapeutic potential.

A recent study demonstrated promising results for phage-antibiotic synergy (PAS) against MDR *K. pneumoniae* in a mouse model, indicating that combining phages with antibiotics could significantly improve therapeutic outcomes [30]. The research showed that phages Pharr (from the *Podoviridae* family) and ФKpNIH-2 (from the *Siphoviridae* family), isolated from wastewater, were effective in reducing bacterial load. Their efficacy was maximized when used alongside antibiotics, suggesting enhanced effectiveness against resistant strains and better infection control. This approach underscores PAS’s potential to target difficult MDR pathogens more robustly than single-agent treatments alone. Such synergy can be especially beneficial for applications like gut decolonization, where localized, non-invasive treatments are preferable. Adding this phage to the antibiotics used in selective digestive decolonization (SDD), that is used in ICU patients to decrease the risk of ventilator-associated pneumonia and other endogenous infections, may potentate the effect of SDD in patients that are colonized with phage-susceptible but multi-drug resistant *K. pneumoniae* strains [31]. This combination approach may also reduce the likelihood of resistance development, as bacteria are exposed to multiple attack mechanisms, lowering their chances of survival and adaptation. Future studies should focus on validating these findings in relevant animal models to assess efficacy, safety, and the dynamics of phage-bacteria interactions in vivo.

## Conclusion

Phage vbKpUKJ_2 shows promising activity against a relevant proportion of non-clonally related, clinical *K. pneumoniae* strains including MDR-strains. Its effective lytic action, lack of antibiotic resistance genes, and genomic stability highlight its potential for therapeutic use. Although vbKpUKJ_2’s host range is somewhat limited, it could serve as a component in a targeted phage therapy approach. This study adds to the literature supporting phages as viable antibiotic alternatives for MDR bacterial infections.

Future research could explore genetic adaptation to broaden its host specificity and conduct in vivo models to further assess vbKpUKJ_2’s therapeutic potential and safety in treating resistant *K. pneumoniae*.

## Electronic supplementary material

Below is the link to the electronic supplementary material.

Supplementary Material 1

Supplementary Material 2

## Data Availability

The genome sequence of bacteriophage vbKpUKJ_2 has been deposited in the NCBI GenBank under the accession number PQ505130 (BankIt2882721 Klebsiella). The data is publicly available.

## Abbreviations

TEM: Transmission electron microscopy
BLAST: Basic local alignment search tool
ONT: Oxford nanopore technology
LSK114: Library preparation kit
PHASTEST: Phage search tool enhanced and speeded
MEGA11: Molecular evolutionary genetics analysis version 11
PFU: Plaque forming units
CFU: Colony forming units
LB: Luria bertani broth
PBS: Phosphate buffered saline
GC: Guanine cytosine
OD: Optical density
NCBI: National center for biotechnology information
MOI: Multiplicity of infection
MDR: Multidrug resistant
ESBL: Extended spectrumβ-lactamase
BMBF: Federal ministry of education and research
DFG: German research foundation
WHO: World health organization
CCD: Charge coupled device
XP: Amplification purification buffer

## References

[1] Ghafourian S, Sekawi Z, Neela V, Khosravi A, Rahbar M, Sadeghifard N. Incidence of extended-spectrum beta-lactamase-producing *Klebsiella pneumoniae* in patients with urinary tract infection. Sao Paulo Med J. 2012;130(1). 10.1590/S1516-31802012000100007 PMC10906682 22344358

[2] Al Bshabshe A, Al-Hakami A, Alshehri B, Al-Shahrani KA, Alshehri AA, Al Shahrani MB et al. Rising *Klebsiella pneumoniae* infections and its Expanding Drug Resistance in the Intensive Care Unit of a Tertiary Healthcare Hospital, Saudi Arabia. Cureus. 2020; Aug 26; 12(8): e10060. 10.7759/cureus.10060 PMC7520404 32999783

[3] Munoz-Price LS, Quinn JP. Deconstructing the infection control bundles for the containment of carbapenem-resistant *Enterobacteriaceae*. Curr Opin Infect Dis. 2013;26(4):378–87. 23806900 10.1097/01.qco.0000431853.71500.77

[4] Tischendorf J, de Avila RA, Safdar N. Risk of infection following colonization with carbapenem-resistant *enterobactericeae*: a systematic review. Am J Infect Control. 2016;44(5):539–43. 26899297 10.1016/j.ajic.2015.12.005 PMC5262497

[5] Shimasaki T, Seekatz A, Bassis C, Rhee Y, Yelin RD, Fogg L, et al. Increased relative abundance of *Klebsiella pneumoniae* carbapenemase-producing *Klebsiella pneumoniae* within the gut microbiota is associated with risk of bloodstream infection in long-term acute care hospital patients. Clin Infect Dis. 2019;68(12):2053–9. 30239622 10.1093/cid/ciy796 PMC6541703

[6] World Health Organization. WHO publishes list of bacteria for which new antibiotics are urgently needed; 2017.

[7] World Health Organization. The advanced HIV disease research landscape. World Health Organization; 2024. Feb 19.

[8] Zhang H, Zhang G, Yang Y, Zhang J, Li D, Duan S, et al. Antimicrobial resistance comparison of *Klebsiella pneumoniae* pathogens isolated from intra-abdominal and urinary tract infections in different organs, hospital departments and regions of China between 2014 and 2017. J Microbiol Immunol Infect. 2021;54(4):639–48. 32247662 10.1016/j.jmii.2020.03.009

[9] Wareth G, Sprague LD, Neubauer H, Pletz MW. *Klebsiella pneumoniae* in Germany: an overview on spatiotemporal distribution and resistance development in humans. German J Microbiol. 2021;1(1).

[10] Gordillo Altamirano FL, Barr JJ. Phage therapy in the postantibiotic era. Clin Microbiol Rev. 2019;32(2):10–128. 10.1128/CMR.00066-18 PMC6431132 30651225

[11] Mirza KA, Nietzsche S, Makarewicz O, Pletz MW, Thieme L. Bacteriophage-mediated decolonization of *Klebsiella pneumoniae* in a novel *Galleria mellonella* gut colonization model with Enterobacteriaceae. Sci Rep. 2024;14(1):318. 38172281 10.1038/s41598-023-50823-9 PMC10764950

[12] Kropinski AM, Mazzocco A, Waddell TE, Lingohr E, Johnson RP. Enumeration of bacteriophages by double agar overlay plaque assay. Bacteriophages: methods and protocols, volume 1: isolation, characterization, and interactions. 2009;69–76. 10.1007/978-1-60327-164-6_7 19066811

[13] Ye Y, Tong G, Chen G, Huang L, Huang L, Jiang X, et al. The characterization and genome analysis of a novel phage phiA034 targeting multiple species of Aeromonas. Virus Res. 2023;336:199193. 37579848 10.1016/j.virusres.2023.199193 PMC10480305

[14] Kolmogorov M, Yuan J, Lin Y, Pevzner PA. Assembly of long, error-prone reads using repeat graphs. Nat Biotechnol. 2019;37(5):540–6. 30936562 10.1038/s41587-019-0072-8

[15] Wishart DS, Han S, Saha S, Oler E, Peters H, Grant JR, et al. PHASTEST: faster than PHASTER, better than PHAST. Nucleic Acids Res. 2023;51(W1):W443–50. 37194694 10.1093/nar/gkad382 PMC10320120

[16] Marquet M, Hölzer M, Pletz MW, Viehweger A, Makarewicz O, Ehricht R, et al. What the phage: a scalable workflow for the identification and analysis of phage sequences. Gigascience. 2022;11:giac110. 36399058 10.1093/gigascience/giac110 PMC9673492

[17] Feldgarden M, Brover V, Gonzalez-Escalona N, Frye JG, Haendiges J, Haft DH, et al. AMRFinderPlus and the reference gene catalog facilitate examination of the genomic links among antimicrobial resistance, stress response, and virulence. Sci Rep. 2021;11(1):12728. 34135355 10.1038/s41598-021-91456-0 PMC8208984

[18] Darling ACE, Mau B, Blattner FR, Perna NT, Mauve. Multiple alignment of conserved genomic sequence with rearrangements. Genome Res. 2004;14(7):1394–403. 15231754 10.1101/gr.2289704 PMC442156

[19] Tamura K, Stecher G, Kumar S. Mol Biol Evol. 2021;38(7):3022–7. MEGA11: Molecular Evolutionary Genetics Analysis Version 11. 10.1093/molbev/msab120 PMC8233496 33892491

[20] Nishimura Y, Yoshida T, Kuronishi M, Uehara H, Ogata H, Goto S. ViPTree: the viral proteomic tree server. Bioinformatics. 2017;33(15):2379–80. 28379287 10.1093/bioinformatics/btx157

[21] Zagui GS, Tonani KAA, Fregonesi BM, Machado GP, Silva TV, Andrade LN, et al. Tertiary hospital sewage as reservoir of bacteria expressing mdr phenotype in Brazil. Brazilian J Biology. 2021;82:e234471. 10.1590/1519-6984.234471 33681897

[22] Edham MH, Al-Tae FMD, Al-Hammadi ATY. Inhibitory effect of bacteriophages isolated from sewage water in the city of Kirkuk on some types of human pathogenic bacteria. Baghdad Sci J. 2017;14(4):0727.

[23] Nazir A, Qi C, Shi N, Gao X, Feng Q, Qing H et al. Characterization and genomic analysis of a Novel Drexlervirial Bacteriophage IME268 with lytic activity against Klebsiella pneumoniae. Infection and drug resistance. 2022:1533–46. 10.2147/IDR.S347110 PMC8994998 35414748

[24] Pacios O, Fernández-García L, Bleriot I, Blasco L, Ambroa A, López M, et al. Phenotypic and genomic comparison of *Klebsiella pneumoniae* lytic phages: vB_KpnM-VAC66 and vB_KpnM-VAC13. Viruses. 2021;14(1):6. 35062209 10.3390/v14010006 PMC8778798

[25] Bleriot I, Blasco L, Pacios O, Fernández-García L, López M, Ortiz-Cartagena C, et al. Proteomic study of the interactions between phages and the bacterial host *Klebsiella pneumoniae*. Microbiol Spectr. 2023;11(2):e03974–22. 36877024 10.1128/spectrum.03974-22 PMC10100988

[26] Donovan DM. Bacteriophage and Peptidoglycan Degrading enzymes with antimicrobial applications. Recent Patents Biotechnol. 2007;1(2):113–22. 10.2174/187220807780809463 19075835

[27] Pirnay JP, Djebara S, Steurs G, Griselain J, Cochez C, De Soir S et al. Personalized bacteriophage therapy outcomes for 100 consecutive cases: a multicentre, multinational, retrospective observational study. Nat Microbiol 2024 4:1–20. 10.1038/s41564-024-01705-x PMC11153159 38834776

[28] Michodigni NF, Nyachieo A, Akhwale JK, Magoma G, Ouédraogo AS, Kimang’a AN. Formulation of phage cocktails and evaluation of their interaction with antibiotics in inhibiting carbapenemase-producing *Klebsiella pneumoniae in vitro* in Kenya. Afr J Lab Med. 2022;11(1):1803. 35937762 10.4102/ajlm.v11i1.1803 PMC9350486

[29] Yoo S, Lee KM, Kim N, Vu TN, Abadie R, Yong D. Designing phage cocktails to combat the emergence of bacteriophage-resistant mutants in multidrug-resistant *Klebsiella pneumoniae*. Microbiol Spectr. 2024;12(1):e01258–23. 38018985 10.1128/spectrum.01258-23 PMC10783003

[30] Hesse S, Malachowa N, Porter AR, Freedman B, Kobayashi SD, Gardner DJ, et al. Bacteriophage treatment rescues mice infected with multidrug-resistant *Klebsiella pneumoniae* st258. MBio. 2021;12(1):10–128. 10.1128/mBio.00034-21 PMC8545083 33622728

[31] Pletz MW, Blasi F, Chalmers JD, Dela Cruz CS, Feldman C, Luna CM, et al. International Perspective on the New 2019 American Thoracic Society/Infectious Diseases Society of America Community-Acquired Pneumonia Guideline: a critical Appraisal by A Global Expert Panel. Chest. 2020;158(5):1912–8. 32858009 10.1016/j.chest.2020.07.089 PMC7445464

