# Supplementary material for: Characterization and genome analysis of novel Klebsiella pneumoniae phage vbKpUKJ_2 isolated from hospital sewage water
Source: BMC Microbiol. 2025 Feb 26;25:96. doi: 10.1186/s12866-025-03813-y (PMC11863478; doi:10.1186/s12866-025-03813-y)
Supplement: Supplementary file 1 — Supplementary Material 1 [file 12866_2025_3813_MOESM1_ESM.pdf]

Supplementary data

Title: Characterization and genome analysis of novel *Klebsiella pneumoniae* phage vbKpUKJ_2 isolated from hospital sewage water.

Authors: Kamran A. Mirza[1,3], Tinatini Tchatchiashvili[1,3], Mike Marquet[1], Sandor Nietzsche[2], Mathias W. Pletz[1,3], Oliwia Makarewicz[1,3]

Affiliations:

[1] Institute of Infectious Diseases and Infection Control, Jena University Hospital, Friedrich-Schiller-University Jena, 07747 Jena, Germany

[2] Center for Electron Microscopy, Jena University Hospital, Friedrich-Schiller-University Jena, 07743 Jena, Germany

[3] Leibniz Center for Photonics in Infection Research, 07747 Jena, Germany

Supplementary Table 1: Results of susceptibility testing for 40 clinical *K. pneumoniae* isolates against phage vbKpUKJ_2, categorized as Susceptible (S), Intermediate susceptible (I), or Resistant (R).

**No. of strains**	***K. pneumoniae* strains ID**	**Phage Susceptibility (S/I/R)**
1	Kp000217	R
2	Kp000413/1	R
3	Kp000796	R
4	Kp000833	I
5	Kp000942	R
6	Kp001030/2	R
7	Kp001049/2	I
8	Kp001079/1	R
9	Kp001109	R
10	Kp001265/2	S
11	Kp001393	R
12	Kp001415	R
13	Kp001453	R
14	Kp001698	R
15	Kp001741/2	S
16	Kp001906	I
17	Kp001946	R
18	Kp001956	I
19	Kp001974	R
20	Kp002092	R
21	Kp002149	R
22	Kp002154	R
23	Kp002308	R
24	Kp002314	S
25	Kp002400	S
26	Kp002423	R
27	Kp00468	R
28	Kp 033335/1	R
29	Kp015026	R
30	Kp033499/1	S
31	Kp0007727	I
32	Kp015073	R
33	Kp033812	R
34	Kp033499/2	S
35	Kp034099/3	S
36	Kp015366	S
37	Kp034238	I
38	Kp419614	S
39	Kp1711_O40741	S
40	ATCC 700603	S

Supplementary Table 2: Genomic screening for antibiotic resistance genes in vbKpUKJ_2, confirming no resistance gene presence.

**Resistance against antibiotics**	**Gene present (yes/no)**
Amikacin	no
Aminocoumarin	no
Aminoglycoside	no

Apramycin	no
Avibactam	no
Avilamycin	no
Azithromycin	no
Bacitracin	no
Beta-Lactam	no
Bleomycin	no
Capreomycin	no
Cefaclor	no
Chloramphenicol	no
Clarithromycin	no
Clindamycin	no
Daptomycin	no
Edine	no
Efflux	no
Erythromycin	no
Fidaxomicin	no
Florfenicol	no
Fosfomycin	no
Fosfomycin G	no
Fusidic Acid	no
G418	no
Gentamicin C	no
Hygromycin	no
Isoniazid	no
Kasugamycin	no
Kirromycin	no
Lincomycin	no
Lincosamide	no
Linezolid	no
Macrolide	no
Maduramicin	no
Methicillin	no
Mupirocin	no
Nalidixic Acid	no
Nansan	no
Neomycin	no
Nitrofurantoin	no
Nitroimidazole	no
Nitroxoline	no
Oleandomycin	no
Osterogrycin	no
Oxazolidinone	no
Paromomycin	no
Phenicol	no
Pleuromutilin	no

Pristinamycin	no
Pulvomycin	no
Quaternary Ammonium	no
Quinolone	no
Retapamulin	no
Rifamycin	no
Rifaximin	no
Salinomycin	no
Spectinomycin	no
Spiramycin	no
Streptogramin A	no
Streptogramin B	no
Streptomycin	no
Streptothricin	no
Sulbactam-Durlobactam	no
Sulfonamide	no
Tamborbactam	no
Tedizolid	no
Telithromycin	no
Temocillin	no
Tetracycline	no
Thiamphenicol	no
Thiostrepton	no
Tiamulin	no
Ticarcillin	no
Ticarcillin-Clavulanic Acid	no
Tobramycin	no
Triclosan	no
Trimethoprim	no
Tylosin	no
Vernamycin B	no
Vancomycin	no
Virginiamycin	no
Virginiamycin M	no
Zoliflodacin	no
Zorbamycin	no
